# Transcranial Direct Current Stimulation and Behavioral Models of Smoking Addiction

**DOI:** 10.3389/fpsyt.2012.00079

**Published:** 2012-08-31

**Authors:** Paige E. Fraser, Allyson C. Rosen

**Affiliations:** ^1^Psychiatry, Palo Alto Veterans Affairs Health Care SystemPalo Alto, CA, USA

**Keywords:** transcranial direct current stimulation, smoking, smoking cessation, repetitive transcranial magnetic stimulation, nicotine

## Abstract

While few studies have applied transcranial direct current stimulation (tDCS) to smoking addiction, existing work suggests that the intervention holds promise for altering the complex system by which environmental cues interact with cravings to drive behavior. Imaging and repetitive transcranial magnetic stimulation studies suggest that increased dorsolateral prefrontal cortex (DLPFC) activation and integrity may be associated with increased resistance to smoking cues. Anodal tDCS of the DLPFC, believed to boost activation, reduces cravings in response to these cues. The finding that noninvasive stimulation modifies cue induced cravings has profound implications for understanding the processes underlying addiction and relapse. tDCS can also be applied to probe mechanisms underlying and supporting nicotine addiction, as was done in a pharmacologic study that applied nicotine, tDCS, and TMS paired associative stimulation to find that stopping nicotine after chronic use induces a reduction in plasticity, causing difficulty in breaking free from association between cues and cravings. This mini-review will place studies that apply tDCS to smokers in the context of research involving the neural substrates of nicotine addiction.

## Introduction

The vast majority of smokers who attempt to quit relapse (CDC, 2008); thus representing an urgent problem in need of additional effective treatments and one in which non-invasive brain stimulation may fill an important niche. Although the reason for this intransigency is a puzzle to neuroscientists, one of the key processes thought to underlie the high rate of relapse is the power of environmental cues to elicit cravings to smoke (Janes et al., 2010; Versace et al., 2011). While current smoking cessation aids are mainly nicotine supplements, there also has been an interest in the impact of brain stimulation on cravings and other correlates of smoking and withdrawal.

The dorsolateral prefrontal cortex (DLPFC) has been a major target of non-invasive stimulation techniques, such as repetitive transcranial magnetic stimulation (rTMS) and transcranial direct current stimulation (tDCS). This brain region is easily accessible to non-invasive stimulation and is believed to exert cognitive control over feelings of craving and reward related to smoking (Goldstein and Volkow, 2011). While the orbitofrontal cortex (OFC) and subcortical regions also are often implicated in smoking addiction, the locations of these structures preclude them as targets for current non-invasive stimulation techniques. Models accounting for the process by which such stimulation modifies cravings focus on controlling functions of the DLPFC itself (McBride et al., 2006; Nestor et al., 2011), as well as possible downstream effects to the subcortical regions involved in the reward system (e.g., Di Chiara, 2000; Haber et al., 2006). tDCS can also be combined with other modalities such as transcranial magnetic stimulation (TMS), functional imaging, and pharmacologic manipulation to explore these multiple, distinct, neurotransmitter systems of reward, and craving. For example, a study characterized a deficit in neuroplasticity induced by lack of nicotine in chronic smokers by applying tDCS, TMS, and pharmacologic challenge (Grundey et al., 2012). Such a deficit of plasticity could be a mechanism underlying the resilience of addiction against therapy, an aspect of smoking cessation that may be possible to address by using the ability of tDCS to modulate plasticity (Nitsche and Paulus, 2000, 2001). This mini-review will place the three, existing, published, studies that apply tDCS to smokers in the context of other studies involving the neural substrates of nicotine addiction to suggest additional future research directions.

## Cognitive Processes Underlying Maintenance Versus Cessation of Smoking Behavior

In smoking research, major subjects of study are craving, drug seeking behavior and related expectations, and relapse. Modulating smoking cue reactivity has been one productive target behavior for brain stimulation (Fregni et al., 2008; Boggio et al., 2009). This approach is accomplished by studying the effect of smoking cues in smokers on self-rated cravings, as smoking cue reactivity is strongly related to smoking relapse (Versace et al., 2011). Neuroimaging studies present smoking cues as stimuli meant to elicit craving responses in smokers, allowing observation of the brain’s reaction. Common cues include video and photographs of people smoking, as well as tactile prompts such as both individual cigarette and pack handling. Reactions to these stimuli are controlled for by exposure to neutral but similar stimuli, such as video or pictures of non-smoking people, handling similarly shaped non-cigarette objects such as pencils. Showing such reward cues even before the rewarding substance in consumed (Childress et al., 1999), as well as vivid cognitive images of reward (Berridge and Robinson, 2003) have been shown to activate the brain regions implicated in mediating reward in the brain.

Whereas most studies on the effect of brain stimulation on smoking focus on response to smoking cues that provoke a craving which can then be modulated, cessation of smoking brings on a second form of craving: abstinence induced craving (Jarvik et al., 2000; Tiffany et al., 2000; Morissette et al., 2005). This feeling is associated with a depletion of nicotine, and is therefore more affected by nicotine than cue induced craving (Tiffany et al., 2000; Morissette et al., 2005). Interestingly, abstinence craving has been shown to be a more effective predictor of relapse than cue induced craving (Killen and Fortmann, 1997; Shiffman et al., 1997). Functional imaging studies show that abstinence craving is associated with activation increases in the right DLPFC and OFC as well as the thalamus (McClernon et al., 2005; Wang et al., 2007) suggesting that they play roles in maintaining abstinence as well as cue induced cravings (Table 1).

**Table 1:** **Dorsolateral prefrontal cortex implication in smoking cue reactivity – studies reporting DLPFC activation in response to smoking cues**.

Author (year)	Imaging technique	Cues	Side	Inc/dec	When
Brody et al. (2002)	PET	Video, cigarette handling	Bi	Inc	With increased craving
Due et al. (2002)*	fMRI	Images	L	Inc	With smoking cues
Franklin et al. (2007)	fMRI	Video, cigarette handling	L	Inc	As craving decreases
Hartwell et al. (2011)*	fMRI	Images	R	Inc	resisting urge to smoke when shown smoking cue
Kober et al. (2010)	fMRI	Images	L	Inc	As craving decreases
Lee et al. (2005)*	fMRI	Images	R	Inc	With smoking cues
McBride et al. (2006)	fMRI	Video	Bi	Inc	With smoking cues in Expectant group
McClernon et al. (2005)*	fMRI	Abstinence	Bi	Inc	With increased craving
Versace et al. (2011)	fMRI	Images	L	Inc	With increased smoking cue reactivity
Wang et al. (2007)	ASL perfusion MRI	Abstinence	R	Inc	With increased craving
Wilson et al. (2005)	fMRI	Cigarette handling	L	Inc	With smoking cues in Expectant group
Wilson et al. (2012)	fMRI	Cigarette handling	L	Inc	With smoking cues
Zhang et al. (2011)	DTI	Images	R	Inc	With smoking cues

*Reported activation in middle frontal gyrus (MFG), which several of these papers note corresponds to DLPFC in Brody et al. (2002), dec, decrease; inc, increase; Bi, bilateral; L, left; R, right; DTI, Diffusion Tensor Imaging; fMRI, functional magnetic resonance imaging; PET, positron emission tomography.

## DLPFC: Stimulation and Imaging

Several imaging studies of smokers have demonstrated that activation in the DLPFC increases in response to seeing smoking cues (Table 1; but, see David et al., 2005). It is generally believed that this activation reflects increased cognitive control (Goldstein and Volkow, 2011). There have been multiple thoughtful and creative paradigms used to elucidate mechanisms underlying how control processes modulate smokers’ response to stimulus cues, including control of motivation, selective attention, working memory, learning, decision making/anticipation, and self-control/behavioral monitoring such as response inhibition. For example, McBride et al. (2006) showed that, even when self-rated craving was equivalent across subjects, the expectation of being able to smoke led to higher DLPFC activation. They concluded that models of control processes of DLPFC should include expectancy and a behavioral system involved in planning and drug seeking.

Because smoking is a known vascular risk (AHA et al., 2012), it is not surprising that neuroimaging studies reveal changes in the structural and functional integrity of the DLPFC that are related to measures of addiction. Long term smoking is associated with decreased gray matter volume of the DLPFC (Brody et al., 2004; Gazdzinski et al., 2005; Gallinat et al., 2006). Studies that performed multimodal imaging (Zhang et al., 2011) showed that this decrease in gray matter density in DLPFC correlated with lower activation of smoker’s brains and greater lifetime exposure to cigarettes. Nestor et al. (2011) found that regardless of the cue type, smokers had less DLPFC activation than both controls and ex-smokers. Lower frontal lobe activation, specifically right superior frontal gyrus (SFG/BA10), during fMRI was associated with higher scores on a measure of nicotine dependence (Fagerström test of nicotine dependence) and more errors on a measure of cognitive control (Go/No-Go). These findings suggest that diminished frontal lobe activation is behaviorally relevant to active smokers. The fact that former smokers did not show this relationship suggests either that there was restoration of frontal lobe functioning after a period of abstinence, or alternatively that successful prefrontal cortex performance facilitated abstinence (Nestor et al., 2011). Thus, increased activation may be a predictor of favorable outcome. Given the evidence that decreased DLPFC integrity is related to smoking history and measures of cognitive dysfunction, this raises the question of whether all smokers could benefit to the same degree from non-invasive brain stimulation. Studies which show that upregulating the frontal lobe improves smoking-related symptoms suggest that even if future studies find that diminished DLPFC ultimately is associated with poorer outcome, these patients may have a greater need for non-invasive stimulation.

High frequency rTMS of the DLPFC, believed to upregulate activation with or without cues, has been shown to both reduce cravings as well as the actual numbers of cigarettes consumed (Table 2). This effect was nearly immediate, with patients showing a significant same day reduction in the number of cigarettes smoked with active rTMS, but not sham controls (Eichhammer et al., 2003). Rose et al. (2011) found similar results stimulating the SFG. The longest-term study, performed by Amiaz et al. (2009) involved 10 days of rTMS treatment to the left DLPFC, followed by an additional month of maintenance. The results showed effects persisting after 6 months, a promising sign for lasting smoking cessation aid.

**Table 2:** **Non-invasive brain stimulation, smoking, and reward – studies reporting the effects of rTMS and tDCS on smoking, related craving, and dopaminergic reward**.

Author (year)	Treatment (days)	MT (%)	Stimulation type	TMS frequency	Stimulated	Side	Cues	Result
**A**								
Amiaz et al. (2009)	10	100	rTMS	10 Hz	DLPFC	L	Images	Reduced cue induced cravings and consumption
Eichhammer et al. (2003)	4 (2 Sham)	90	rTMS	20 Hz	DLPFC	L	None	Reduced cigarette consumption
Johann et al. (2003)	2	90	rTMS	High	DLPFC		None	Reduced craving
Rose et al. (2011)	3	90	rTMS	10 Hz	SFG	L	View lit cigarette while handling cigarette and lighter	Increased cue induced craving, reduced general craving
						L	Cigarette smoke	Reduced craving
Soo Cho and Strafella (2009)*	1	100	rTMS	10 Hz	DLPFC	L	None	DA release in ipsilateral ACC and mOFC
						R	None	None
Strafella et al. (2001)*	1		rTMS	10 Hz	DLPFC	L	None	DA release in ipsilateral caudate nucleus
Strafella et al. (2003)*	1	90	rTMS	10 Hz	M1	L	None	DA release in ipsilateral caudate nucleus
**B**								
Boggio et al. (2009)	5		tDCS		DLPFC	L – anodal R – cathodal (reference)	Video, cigarette handling	Decreased cue induced craving
Fregni et al. (2008)	1		tDCS		DLPFC	L – anodal R – cathodal (reference)	Video, cigarette handling	Decreased cue induced craving
						R – anodal L – cathodal (reference)	Video, cigarette handling	Decreased cue induced craving
Grundey et al. (2012)	1		tDCS		ADM (orbit as reference)	ADM – anodal Orbit – cathodal	Abstinence	Control: no significant increase, with nicotine: increased excitability
						Orbit – anodal ADM – cathodal	Abstinence	Control: reduced excitability, with nicotine: effects abolished

(A) Studies reporting the effects of rTMS on smoking or related craving, or the effects on dopamine release, *, not studied in smokers; L, left; R, right; ACC, anterior cingulate cortex; DA, dopamine; M1, motor cortex; MT, motor threshold; mOFC, medial orbitofrontal cortex; SFC, superior frontal gyrus. (B) Studies of the effects of tDCS on smokers; ADM, motor cortex representational area of the abductor digiti minimi muscle.

The success of these rTMS studies served as a basis for targeting DLPFC with tDCS (Table 2). Thus far there have been two studies that apply tDCS to modulate frontal lobe activity in smokers, and they have yielded promising results. In the study by Fregni et al. (2008) subjects were given randomized active or sham tDCS in conjunction with video and cigarette handling. A single anodal tDCS session over either left or right DLPFC (with cathodal stimulation on contralateral DLPFC) significantly reduced the self-reported craving levels elicited by these cues, with no significant mood changes. Furthermore, these effects were dose dependent, such that repeated sessions led to an increasingly powerful response (Boggio et al., 2009). In fact, by the end of Boggio et al.’s 5 day stimulation course, the group receiving active tDCS not only showed reduced craving ratings, but were also observed to smoke at least 30% fewer cigarettes per day, demonstrating a clinically significant effect on smoking cessation.

## tDCS as a Measure of Nicotine Effects on Plasticity

One process underlying nicotine addiction may involve diminished neuroplasticity induced by an absence of nicotine after chronic use. This diminished plasticity during the withdrawal state may be an important barrier to smoking cessation, as it limits the ability of the brain to decouple the pairing between environmental cues and cravings. Nicotine influences many systems known to be involved in generating and modulating plasticity. In addition to the dopaminergic system, it affects the nicotinic acetylcholine receptors (nAChRs), as well as the adrenergic, serotonergic, glutamatergic, and GABAergic systems (Levin et al., 2006).

Because tDCS has been shown to modulate plasticity (Nitsche and Paulus, 2000, 2001), Grundey et al. (2012) applied stimulation to study the deficit of neuroplasticity associated with withdrawal. They combined paired associative stimulation (PAS), paired pulse TMS paradigm that modulates plasticity, with tDCS which can amplify these effects, to study the effect of nicotine on plasticity in smokers. In a PAS paradigm, peripheral nerve stimulation (right ulnar nerve at the wrist level) was followed by a single-pulse of low frequency TMS to the motor cortex. Depending on the interpulse interval between the two types of stimulation, the excitability of the motor evoked potential (MEP, the size of the TMS induced muscle contraction) increased (facilitation with 25 ms interpulse interval) or decreased (excitability diminishing with 10 ms interpulse interval) with repeated pairings (Stefan et al., 2000; Wolters et al., 2003). Under conditions of normal plasticity, tDCS should augment the effects of PAS with anodal tDCS increasing PAS facilitation and the cathodal tDCS reducing further the PAS excitability diminution. For smokers in withdrawal (10 h of abstinence), anodal tDCS to the motor cortex representational area of the right abductor digiti minimi muscle (ADM), using the area above the right orbit as a reference, did not significantly augment PAS facilitation, but with the addition of nicotine, there was normalization of the system such that anodal stimulation yielded a significant enhancement of excitability for hours after the stimulation was administered. Conversely, in the withdrawal condition, cathodal tDCS produced a significant decrease in excitability that was nearly abolished with the administration of nicotine. This suggests that for abstinent smokers, nicotine compensates for a deficit in plasticity which can be studied with tDCS.

## Future Directions and Unanswered Questions

Whereas DLPFC stimulation shows promise in reducing the power of smoking-related cues to elicit craving, brain imaging before and after therapy (e.g., MR connectivity, PET ligand studies of receptor changes) enables characterization of the network of connections between brain regions that may be indirectly altered. Affected regions might include areas involved in smoking cue reactivity such as the visual association cortex, dorsal striatum, anterior cingulate cortex, prefrontal cortex and insula, and likely the nucleus accumbens (Versace et al., 2011). Localized stimulation from both TMS and rTMS has been shown to have non-local effects (George et al., 1999; Kimbrell et al., 2002), but can only indirectly reach subcortical structures. Because tDCS involves current flow between the anodal and cathodal components, it is not known whether additional brain structures, such as the OFC which has connections to the amygdala and striatum – structures involved in mediating predictive reward value (O’Doherty, 2003) – could be directly reached with tDCS or whether the combination of both upregulation of one DLPFC and downregulation of the contralateral DLPFC can function synergistically as has been observed with tDCS of DLPFC in risk taking (Fecteau et al., 2007).

Combining pharmacologic antagonists with brain stimulation is a powerful approach for studying the neurochemical substrates of the treatment benefits of tDCS. For example, studies imaging the brain with PET ligands both before and after TMS support a model in which DLPFC stimulation indirectly modulates the brain’s reward system. Strafella et al. (2001) applied rTMS to the DLPFC and showed dopamine release in brain regions implicated in addiction, notably the ipsilateral caudate nucleus, and medial prefrontal cortex, including pre- and sub-genual anterior cingulate cortex, and medial OFC, all areas implicated in addiction (Table 2; Soo Cho and Strafella, 2009). Eichhammer et al. (2003) posit that using TMS to increase DLPFC activation may mimic reward, and Fregni et al. (2008) suggest that stimulation with tDCS may have similar effects. This hypothetical mechanism is corroborated by a study done by Nitsche et al. (2006) showing that an antagonist that blocks the D2 receptors almost completely negates the excitability diminishing after-effects of cathodal tDCS, suggesting that dopamine receptor activation may control the induction of tDCS generated excitability. In addition to studying the connections involved, it is advantageous to have anatomical information regarding the locus of brain changes, as direct surgical stimulation of these regions might prove useful in patients where their addiction has life threatening consequences such as risk of stroke.

The rTMS, tDCS, and fMRI studies reviewed here suggest that increasing DLPFC activity should reduce craving; however, a stimulation study of craving modulation in abstinent smokers has yet to be performed. The work in Grundey et al.’s (2012) reveals that patients in withdrawal should be considered separately from those actively smoking due to a deficit in plasticity stemming from the removal of nicotine. Thus, the nicotine maintenance status of these individuals should be carefully tracked. Additionally, this lack of plasticity should be taken into consideration in all therapies involving smokers, as patients without nicotine supplementation may not benefit from therapies that depend on the form of memory that is probed by the paired associate stimulation paradigm. However, stimulation performed in conjunction with supplemental nicotine may reinstate plasticity, giving patients the ability to dissociate cue/craving pairings, and reducing the power of the cues to evoke relapse. Therefore, the therapeutic effects of tDCS could be augmented by pharmacologic intervention and combination therapies such as nicotine administration.

Furthermore, identification of multiple, distinct brain systems that mediate rewards and craving may elucidate the mechanisms by which DLPFC stimulation alters cue responsiveness. Models from the animal literature has shown that the reward system can be separated into two distinct processes; the dopaminergic “wanting” (seeing incentives as desirable compared with other stimuli) and the opiate and GABAergic “liking” (linked to conscious pleasure) pathways (Wyvell and Berridge, (2000; Reynolds and Berridge, 2002; Berridge and Robinson, 2003). Because nicotine has effects in both the “wanting” and “liking” systems of reward (Levin et al., 2006), it is possible that paired pulse paradigms could be used to probe these disparate neurotransmitter systems as Di Lazzaro et al. (2007) did by using to PAS in combination with GABA type A receptor (GABAAR) modulating drugs to differentiate two GABAAR subtypes. Understanding the roles these reward systems play in addiction may be extremely valuable in the creation of more effective smoking treatments.

Finally, there are likely additional approaches for augmenting the tDCS treatment effects, such as manipulating the state in which the stimulation occurs, as studies of state dependency demonstrate effects on stimulation outcome (e.g., Silvanto et al., 2007, 2008; Silvanto and Pascual-Leone, 2008;). One of the critical problems for therapies is generalization. While treatments may reduce craving in the office, patients often relapse at home; the finding by Boggio et al. (2009) demonstrating reductions in cigarettes smoked after tDCS is thus compelling. However, unlike rTMS, tDCS is portable, and can therefore be delivered in the home. It remains to be seen whether stimulation under the influence of the patient’s natural state of cues in their habitual smoking environment could enhance this benefit. Assessing stimulation induced reductions in smoking cue responsivity in the environment where much of their smoking behavior occurs may also be a more sensitive predictor of treatment response than similar evaluations in the lab, allowing the duration and dose of therapy to be appropriately adapted. Pragmatically, in treating smokers for whom daily TMS sessions in a clinic are not feasible, this in-home stimulation may also reach more patients. Additionally, used in conjunction with portable, mobile devices to measure psychophysiology (e.g., heart rate variability, digital palmar temperature) researchers and clinicians can move beyond the laboratory based cues into those found in patients’ naturalistic settings. These devices promise to provide further clues about reactivity to stimuli in the patient’s everyday life, monitoring physiological reactions to spousal conflict or other sources of acute stress, which have been shown to increase cigarette craving (Childs and de Wit, 2010). This possibility for *in vivo* study would be especially powerful if used in conjunction with longitudinal fMRI sessions with images from the patients’ own environment to track changes in the neural substrates of behavior over the course of treatment. Thus, tDCS presents an opportunity to study and address several disparate barriers to smoking cessation *in vivo*: smoking cue induced craving, abstinence induced craving, withdrawal-induced neuroplasticity deficits, and the involvement of reward subtypes.

## Conflict of Interest Statement

The authors declare that the research was conducted in the absence of any commercial or financial relationships that could be construed as a potential conflict of interest.
